# Are Postgraduate Medical Residency Training Positions in Atlantic Canada Evenly Distributed?

**DOI:** 10.7759/cureus.574

**Published:** 2016-04-17

**Authors:** Paul Atkinson, Mike Howlett, Jacqueline MacKay, Jacqueline Fraser, Peter Ross

**Affiliations:** 1 Emergency Medicine, Saint John Regional Hospital; 2 Emergency Medicine, Dalhousie University; 3 Emergency Medicine, Saint John Regional Hospital / Dalhousie University; 4 Family Medicine, Saint John Regional Hospital / Dalhousie University; 5 Emergency Medicine, Horizon Health Network

**Keywords:** resident training, workforce planning, distributed education

## Abstract

Background

The distribution of postgraduate medical training (residency) positions in Canada is administered by medical schools and universities in conjunction with individual provinces. In Atlantic Canada, the Maritime provinces are considered a single unit under Dalhousie University in Nova Scotia (NS), although distributed medical undergraduate education through Dalhousie and Sherbrooke has enabled medical students to complete their entire course of study in New Brunswick (NB). It is unclear if postgraduate medical education has been distributed in a similar fashion in Atlantic Canada, particularly in New Brunswick and Prince Edward Island (PE).

Methods

Data on the number of R1 residency positions was obtained from the Canadian Resident Matching Service (CaRMS) database. The distribution of R1 positions was described and compared nationally and through the Atlantic provinces. The analysis was completed using MS Excel and Prism.

Results

Rates of R1 positions per million persons varied widely; the national median rate was 97 positions per million persons, with a range of 34 to 138. The combined Maritime provinces rate of R1 positions was 71 per million persons and the rate in Newfoundland (NL) was 138 positions per million. The NS rate was 106 positions per million while the NB rate was 54 per million and the PE rate 34 per million. Sixty-four percent of all residency training positions in Atlantic Canada were based in the two most urban areas of Halifax, NS or St John’s, NL. Royal College (specialty) positions were more likely to be based at the main university campus city than family medicine training positions (97 vs. 3%; 33 vs. 67%, respectively).

Conclusion

There is a high level of variation in available residency positions among the individual provinces, especially in Atlantic Canada. The lower prevalence of opportunities in NB and PE may influence the ability of these provinces to recruit and retain new physicians.

## Introduction

In Canada, individual provinces and universities determine the size of medical school classes, the number of residencies, and how the training positions are divided between specialties. There is no national system to analyze and adapt training numbers based on population demographics that might project a future requirement for physicians [1]. The Canadian Resident Matching Service (CaRMS) lists all available residency positions in Canada [2]. The four provinces in Atlantic Canada (Newfoundland and Labrador, NL; Nova Scotia, NS; New Brunswick, NB; Prince Edward Island, PE) are served by two Universities; Dalhousie and Memorial University, as well as a distributed program from Sherbrooke University, Quebec, based in Moncton, NB. For residency application, CaRMS lists the Maritime provinces (NS, NB, and PE) as a single unit, under Dalhousie University. This highlights that New Brunswick and Prince Edward Island are the only Canadian provinces that do not have their own medical school. Dalhousie University has recently expanded its undergraduate program to include a site in New Brunswick – Dalhousie Medicine New Brunswick (DMNB) in Saint John, and Sherbrooke has a satellite program at the Université de Moncton. We wished to determine if postgraduate medical training is distributed in a similar fashion in Atlantic Canada, and especially in NB and PE, and to compare rates of resident positions with the rest of the country.

Our primary question was” Do provinces that lack a medical school have lower rates of R1 residency positions than other provinces?”

Our secondary question was “Is the ratio of residency training positions to population evenly distributed in Atlantic Canada?”

## Materials and methods

Data on the number of R1 residency positions available in 2015, including the university and the listed home base city or region, were abstracted from the CARMS database [2]. Population statistics for each province in 2015 were obtained from Statistics Canada [3].

We described the distribution of available R1 residency positions nationally and within Atlantic Canada (Dalhousie and Memorial Universities) and compared provincial numbers of R1 positions according to the population served.

Data was analyzed using MS Excel (Microsoft Corp) and Prism (Graphpad Software) using Fisher’s Exact and Wilcoxon Signed Rank Tests.

## Results

In 2015, CaRMS listed 3,321 R1 residency positions across Canada. When the Maritime provinces are combined as a single center, rates of residency positions with respect to population (per million persons) were similar across Canada. Rates varied from 72 per million in British Columbia (BC) to 138 per million in Newfoundland and Labrador (NL), with a combined Maritime provinces rate of 71 per million.

**Figure 1:**
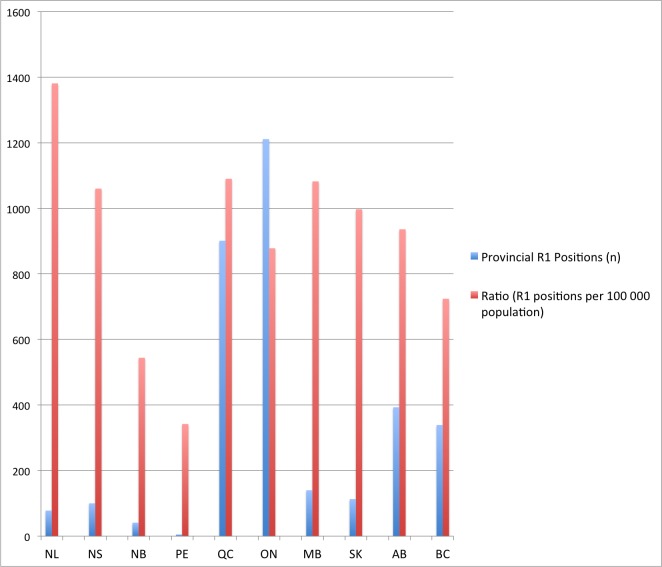
Number and rates of R1 residency positions by province in 2015. Note: Rate of R1 residency positions shown per 100,000 population to optimize visualization.

When the Maritime provinces (NS, NB, and PE) were considered as separate training centers, we found that the variation in rates of R1 positions per million persons increased. The national median rate was 97 R1 positions per million persons, with a range of provincial rates of 34 to 138. The rate in Nova Scotia remained close to the national median at 106, whereas the rates in New Brunswick and Prince Edward Island were 54 and 34, respectively. These provincial rates were significantly lower than the national median (p < 0.01; Wilcoxon Signed Rank Test) and fall below the 25th percentile (63 per million). These results are reported in Table 1 and Figure 1.

**Table 1:** Number and Rates of R1 Residency Positions by Province in 2015

Province	R1 Residency Positions	Population (Millions)	Ratio R1 per Million Population
NL	78	0.565	138
NS	100	0.943	106
NB	41	0.754	54
PE	5	0.146	34
QC	901	8.26	109
ON	1211	13.79	88
MB	140	1.29	108
SK	113	1.13	100
AB	393	4.20	94
BC	339	4.68	72
Canada (Total/median)	3,321	35.77	97

**Figure 2:**
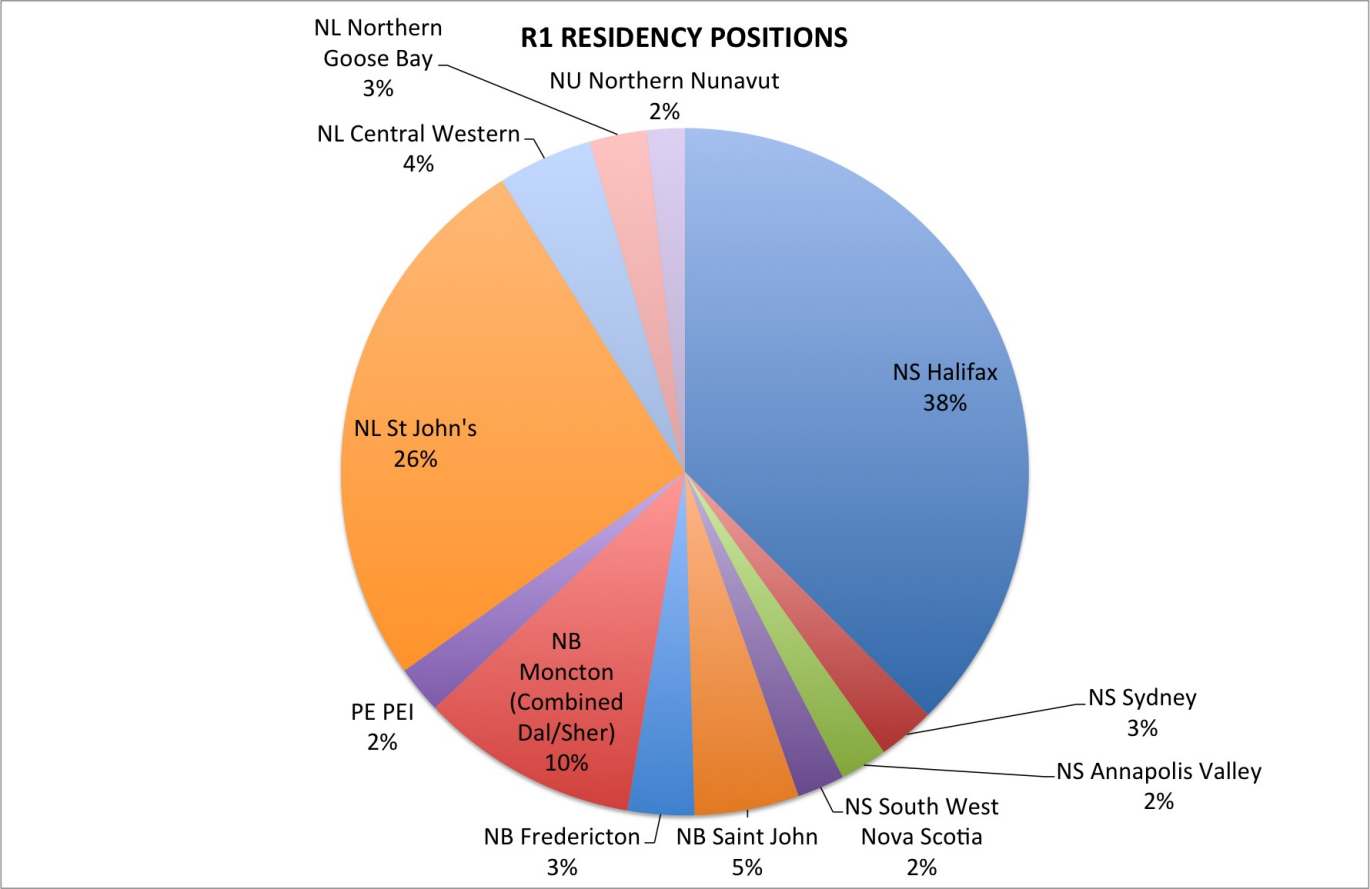
Distribution of R1 residency positions in Atlantic Canada in 2015.

There were 224 R1 residency positions in Atlantic Canada with 130 at Dalhousie, 78 at Memorial, and 16 at Sherbrooke (Moncton) Universities. When the distribution of R1 positions within Atlantic Canada was analyzed, these positions were found to be clustered around cities where the main medical school campus is found. Overall, 64% of all residency training positions in Atlantic Canada were based in Halifax, NS (84 positions) or St John’s, NL (58 positions). Detailed numbers by city or region are reported in Table 2 and Figure 2.

**Table 2:** Distribution of R1 Residency Positions in Atlantic Canada in 2015

Province/University	Home Base	R1 Residency Positions
NS/Dalhousie	Halifax	84
NS/Dalhousie	Sydney	6
NS/Dalhousie	Annapolis Valley	5
NS/Dalhousie	South West Nova Scotia	5
NB/Dalhousie	Saint John	11
NB/Dalhousie	Fredericton	7
NB/Dalhousie	Moncton	7
NB/Sherbrooke	Moncton	16
PE/Dalhousie	PEI	5
NL/Memorial	St John's/Eastern	58
NL/Memorial	Central Western	10
NL/Memorial	Northern Goose Bay	6

Royal College specialty positions were more likely to be based at the main university campus city (Halifax or St. John’s) than family medicine training positions (97 vs. 3%; and 33 vs. 67%, respectively; p < 0.0001; Fisher’s Exact Test) (Table 3).

**Table 3:** Comparison of Home or Base Location for Specialty and Family R1 Residency Positions for Atlantic Canadian University Postgraduate Training Programs

		Main Campus (Halifax/St. John's)	Other Location	Total
Dalhousie U.	Royal College / Specialty	67	4	71
	Family	17	42	59
Memorial U.	Royal College / Specialty	45	0	45
	Family	13	20	33
Combined	Royal College / Specialty	112	4	116
	Family	30	62	92

## Discussion

Our data shows that Canadian provinces without a main campus medical school have lower rates of R1 residency positions than other provinces. This is seen in the Atlantic provinces of New Brunswick and Prince Edward Island, which have the lowest rates of R1 residency positions of all Canadian provinces (54 and 34 per million, respectively). In addition to having lower rates of training positions,  provinces lacking their own medical schools host mainly generalist family medicine positions, with Royal College specialty training positions largely being located close to the main medical school campuses. Within Atlantic Canadian programs, the clustering of all available R1 positions in cities where the main medical school campus is based is similar at Dalhousie University in Halifax (67%) and at Memorial University in St. John’s (74%).

Within the Dalhousie University program, Nova Scotia has 77%, New Brunswick has 19%, and Prince Edward Island has only 4% of all R1 (first year) postgraduate training positions. This is likely to translate to less specialists and family doctors for NB and PE, as they will be established elsewhere. Previous findings from distributive programs have shown that residents in both specialty and family medicine programs were more likely to end up working as physicians in a region, if they had spent time training there [4]. Only four specialist Royal College residency positions were based outside of Nova Scotia (Internal Medicine in Saint John, NB). A similar pattern of clustering of specialist Royal College residency positions around the main medical school campus was seen in the Memorial program. 

It is likely that there are factors beyond population demographics influencing this uneven distribution. Historically, postgraduate training has been centred around university hospitals. This clustering of Royal College specialty residency positions around main campus locations was confirmed by our findings and may reflect trends seen nationally. This poses a major challenge for provinces, such as NB and PE, who have tertiary hospitals providing specialty services but without residency training programs to support and sustain these services. Overall, these figures may explain the reported difficulty that some provinces have with recruitment of both general and specialist physicians.

It is clear that provinces without medical schools will have greater challenges with future recruitment. It could be argued that combined regional postgraduate training programs do not distribute their trainees equitably. Factors, such as available expertise and government funding, are likely to contribute to this effect.

What solutions might be feasible? The principles of population based funding indicate that resources should be directed toward the areas of greatest need. Distributed programs offer training positions and rotations at more community and rural-based locations and have been trialed in New Brunswick. Should universities that are funded by more than one province, such as Dalhousie and Memorial, work toward the improved distribution of postgraduate programs according to population and needs, and not cluster them around the main campus? Should postgraduate training be aligned with undergraduate training using a similar distributive model? Or should the provincial governments in New Brunswick and Prince Edward Island look to develop their own medical schools designed to meet the needs of the local population?

## Conclusions

Postgraduate residency training opportunities in Canada are allocated by province and university program location. However, the Maritime provinces are considered as a single training region and are shown to demonstrate a high level of variation between individual provinces for available residency positions. The reported lower prevalence of opportunity in New Brunswick and Prince Edward Island may lead to reduced recruitment and retention of new physicians and could be adjusted using distributive models based on a population and needs assessment.

## Appendices

### Appendix 1. Canadian Provinces and Territories Two-Letter Abbreviations

Alberta AB

British Columbia BC

Manitoba MB

New Brunswick NB

Newfoundland and Labrador NL

Northwest Territories NT

Nova Scotia NS

Nunavut NU

Ontario ON

Prince Edward Island PE

Quebec QC

Saskatchewan SK

Yukon YT
